# Supplementary material for: C/EBPɑ is crucial determinant of epithelial maintenance by preventing epithelial-to-mesenchymal transition
Source: Nat Commun. 2020 Feb 7;11:785. doi: 10.1038/s41467-020-14556-x (PMC7005738; doi:10.1038/s41467-020-14556-x)
Supplement: Supplementary file 2 — Supplementary Information [file 41467_2020_14556_MOESM2_ESM.pdf]

Supplementary information

C/EBPα is crucial determinant of epithelial maintenance by preventing epithelial-to-mesenchymal transition

Lourenço, et al.

Supplementary Figures

Supplementary Figure 1. *CEBPA* but not *CEBPB* is downregulated during TGF-β-mediated EMT
Supplementary Figure 2. 355 genes are commonly regulated by C/EBPα and TGF-β
Supplementary Figure 3. C/EBPα overexpression impacts cell migration but not cell growth
Supplementary Figure 4. *CEBPA* knock-down induces EMT in both HMLE and MCF10A cells
Supplementary Figure 5. The effect of CEBPA knock-down on EMT-effectors
Supplementary Figure 6. The impact of doxycycline-inducible CEBPA on MCF10A-derived epithelial-spheroids treated with TGF-β
Supplementary Figure 7. C/EBPα expression levels in the tumor organoids and primary tumors
Supplementary Figure 8. Clinical relevance of C/EBPα expression in breast cancer
Supplementary Figure 9. Western blot results of the effect of TGF-β treatment on HMLE cells
Supplementary Figure 10. Western blot results of the effect of C/EBPα overexpression on TGF-b-mediated EMT
Supplementary Figure 11. Western blot results of the effect of C/EBPα knockdown on EMT markers in HMLE cells
Supplementary Figure 12. Western blot results of the effect of C/EBPα conditional activation on EMT
Supplementary Figure 13. Western blot results of the effect of C/EBPα overexpression on Smad3 activity during TGF-b treatment
Supplementary Figure 14. Western blot results of the effect of C/EBPα knockdown on EMT markers in MCF10A cells
Supplementary Figure 15. Western blot results of the effect of C/EBPα conditional activation on EMT in MCF10A cells

Supplementary Figures

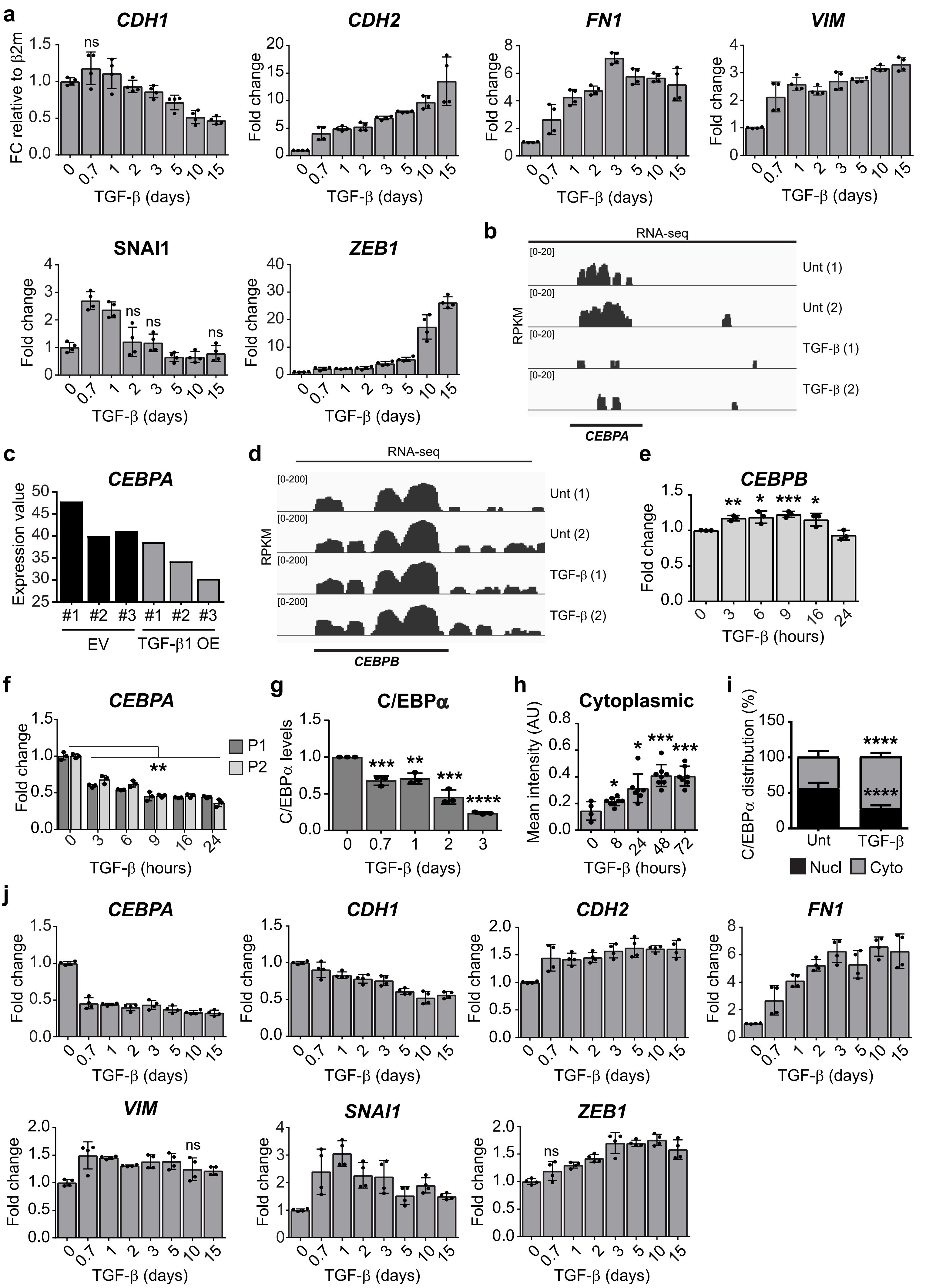

Supplementary Figure 1. *CEBPA* but not *CEBPB* is downregulated during TGF-β-mediated EMT. (a) qRT-PCR analysis showing the effect of TGF-β on several well-known EMT mesenchymal and epithelial markers in HMLE cells. Fold change relative to beta-2 microglobulin. Data represented as mean ± SD of four independent experiments. Statistical significance was calculated using unpaired two-tailed Student's t-test. $p<0.05$ except when indicated with ns (not significant). **(b)** Visualization of RNA-seq profile for the CEBPA locus in TGF-β treated and untreated HMLE cells in both duplicates. **(c)** Analysis of CEBPA expression in control or TGF-β1-overexpressing HMLE cells from public microarray data available from Taube et al. (Accession number GSE24202). Results from 3 replicates per group. **(d)** Visualization of RNA-seq profile for the CEBPB locus in TGF-β treated (24 hours) and untreated HMLE cells in both duplicates. **(e)** qRT-PCR results showing the effect of TGF-β on *CEBPB* mRNA levels. Fold change relative to beta-2 microglobulin. Data represented as mean ± SD of three independent experiments. Statistical significance was calculated using unpaired two-tailed Student's t-test. *$p<0.05$, **$p < 0.01$ and ***$p < 0.001$. ns: not significant. **(f)** qRT-PCR results showing the effect of TGF-β on *CEBPA* mRNA levels at shorter time points. Fold change relative to beta-2 microglobulin. Two independent set of primers were used (P1 and P2). Data represented as mean ± SD of three independent experiments. p-values were calculated using unpaired two-tailed Student's t-test. **$p < 0.01$.**(g)** Quantification of C/EBPα protein levels upon short TGF-β treatment and normalized to tubulin of three independent experiments. p-values were calculated using unpaired two-tailed Student's t-test. **$p < 0.01$, ***$p < 0.001$ and ****$p < 0.0001$. **(h)** HMLE cells were treated with TGF-β for short time points as indicated and immunofluorescence staining was performed to assess cellular localization of C/EBPα. Quantification of C/EBPα cytoplasmic signal normalized to the total area is shown. Data represented as mean ± SD of n=4-8 independent experiments. p-values were calculated using unpaired two-tailed Student's t-test. *$p < 0.05$ and ***$p < 0.001$. **(i)** HMLE cells were treated with TGF-β for 15 days and immunofluorescence staining was performed to assess C/EBPα expression levels and cellular localization. Quantification of the percentage of area occupied by C/EBPα in the nucleus (Nucl) and cytoplasm (Cyto) is shown. Data represented as mean ± SD of four-five independent experiments. p-values were calculated using unpaired two-tailed Student's t-test. ****$p < 0.0001$. **(j)** MCF10A cells were either left untreated or treated with 5 ng/ml of TGF-β as indicated. qRT-PCR analysis showing the effect of TGF-β on *CEBPA* and well-known EMT markers. Fold change relative to beta-2 microglobulin. Data represented as mean ± SD of four independent experiments. Statistical significance was calculated using unpaired two-tailed Student's t-test. $p<0.05$ except when indicated with ns (not significant).

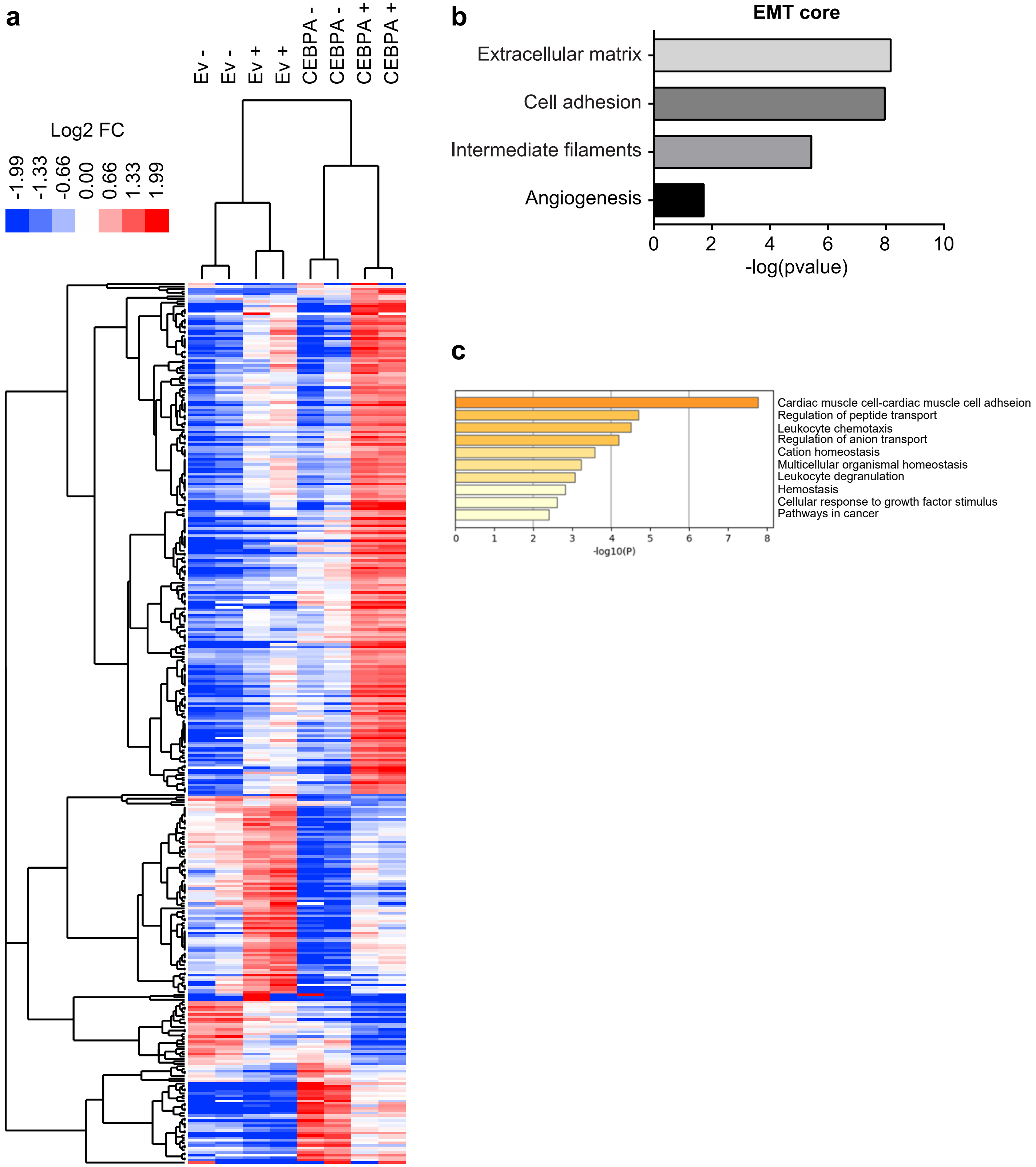

Supplementary Figure 2. 355 genes are commonly regulated by C/EBPα and TGF-β. (a) Heatmap visualizing gene expression of the 355 TGF-β- and C/EBPα-regulated genes. **(b)** Gene ontology (GO) analysis on the EMT core genes. **(c)** GO-Term enrichment analysis of the 20 genes comprising cluster 2 and which expression is increased in C/EBPα-overexpressing cells in both untreated and TGF-β treated conditions. Metascape software was used for this analysis.

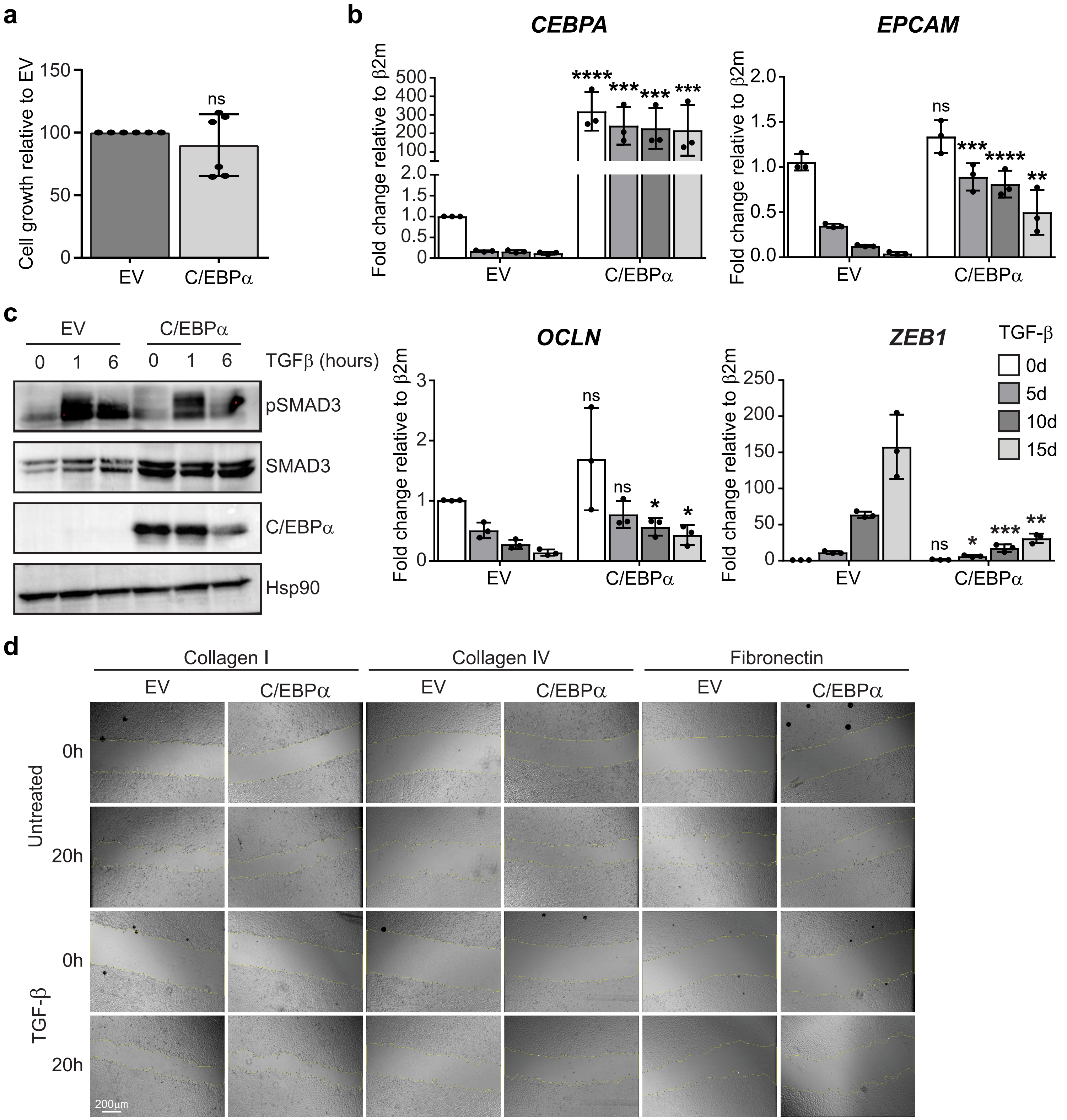

Supplementary Figure 3. **C/EBPα overexpression impacts cell migration but not cell growth. (a)** Graph showing percentage of cell growth of C/EBPα-expressing HMLE cells compared to control EV HMLE cells. Data represented as mean ± SD of six independent experiments. Statistical significance was calculated using unpaired two-tailed Student's t-test (ns: not significant). **(b)** Results from qRT-PCR analysis showing *CEBPA*, *EPCAM*, *OCLN* and *ZEB1* mRNA levels in HMLE cells expressing empty vector or constitutive CEBPA, untreated or TGF-β treated as indicated. Data represented as mean ± SD of three independent experiments. p-values were calculated using unpaired two-tailed Student's t-test. $*p < 0.05$, $**p < 0.01$, $***p < 0.001$ and $****p < 0.001$. ns: not significant. **(c)** HMLE cells expressing empty vector (EV) or constitutive CEBPA were either left untreated or treated with 2.5ng/ml of TGF-β for 0, 1 or 6 hours. Immunoblotting analysis for the indicated proteins were performed. Data is representative of three independent experiments. **(d)** Representative images of wound-healing assays performed using EV or C/EBPα-overexpressing HMLE cells, treated for 20h with or without TGF-β (2.5ng/ml). Studies were performed on coated wells using three different components of the extracellular matrix. Data is representative of n=18-24 independent experiments. Scale bar: 200µm.

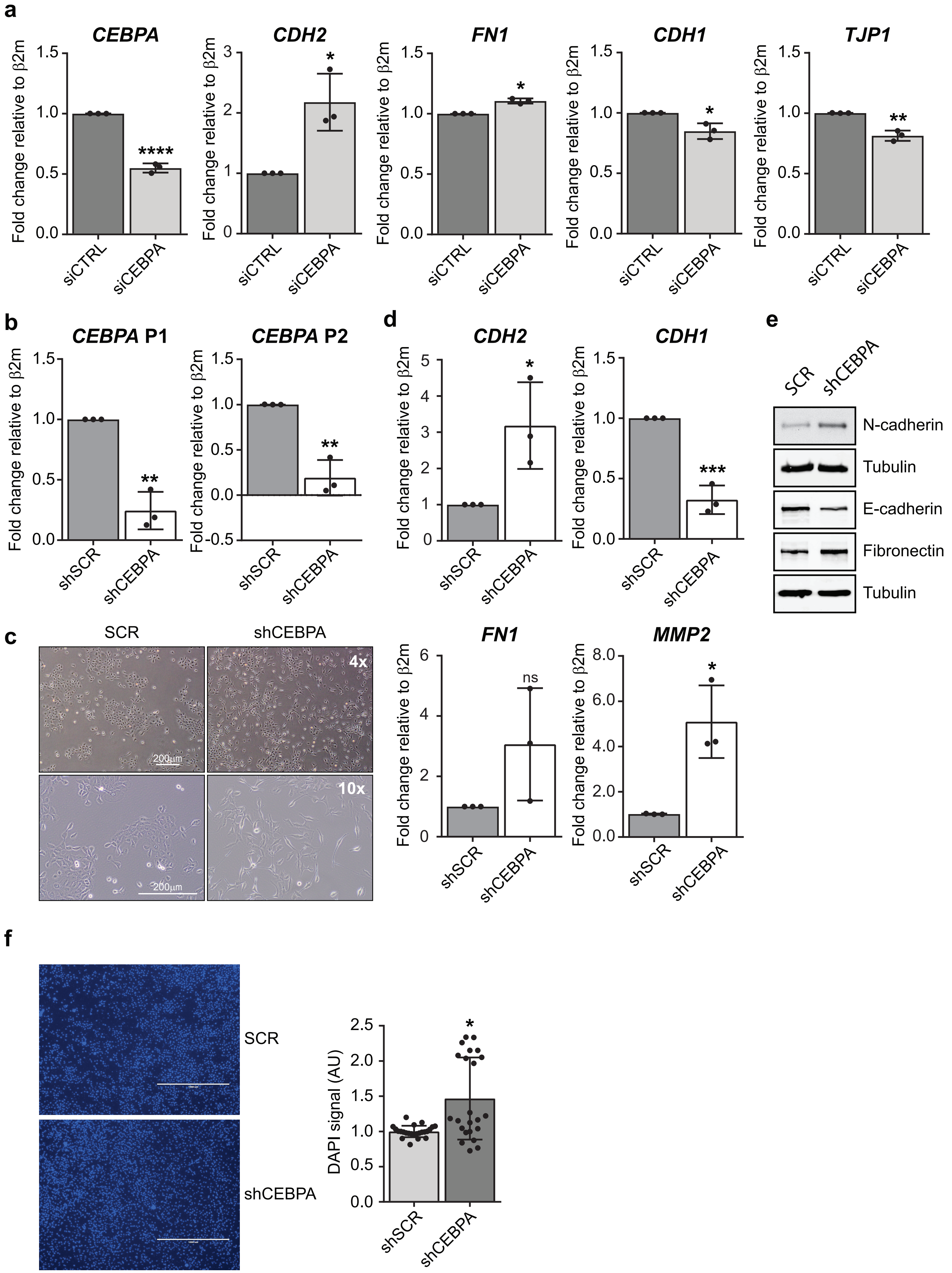

a
CEBPA
CDH2
FN1
CDH1
TJP1
Fold change relative to β2m
siCTRL
siCEBPA
****
*
**
b
CEBPA P1
CEBPA P2
shSCR
shCEBPA
d
MMP2
ns
***
e
SCR
shCEBPA
N-cadherin
Tubulin
E-cadherin
Fibronectin
c
4x
10x
200μm
f
DAPI signal (AU)

Supplementary Figure 4. *CEBPA* knock-down induces EMT in both HMLE and MCF10A cells. **(a)** *CEBPA*, *CDH2*, *FN1*, *CDH1* and *TJP1* mRNA levels in HMLE cells transfected with siRNA SMARTpool targeting *CEBPA*. Data represented as mean ± SD of three independent experiments. p-values were calculated using unpaired two-tailed Student's t-test. *$p < 0.05$, **$p < 0.01$ and ****$p < 0.0001$. **(b)** *CEBPA* mRNA levels in MFC10A cells expressing shRNA control or shRNA targeting CEBPA. Two independent set of primers were used (P1 and P2). Data represented as mean ± SD of three independent experiments. p-values were calculated using unpaired two-tailed Student's t-test. **$p < 0.01$. **(c)** Bright-field microscopy images of MFC10A cells expressing shRNA control (SCR) or shRNA targeting *CEBPA* in two different magnifications. Data is representative of three independent experiments. Scale bar: 200µm. **(d)** qRT-PCR data showing the expression levels of known epithelial and mesenchymal markers upon *CEBPA* knock-down in MCF10A cells. Data represented as mean ± SD of three independent experiments. p-values were calculated using unpaired two-tailed Student's t-test. *$p < 0.05$ and ***$p < 0.001$. ns: not significant. **(e)** Results from immunoblotting analysis displaying protein levels of well-characterized EMT markers in the presence of *CEBPA* knock-down. Data is representative of three independent experiments**. (f)** Representative images of transwell assays stained for DAPI to visualize migrated MCF10A control cells (shSCR) or *CEBPA*-knock-down MCF10A cells (shCEBPA). Data is representative of n=24 independent experiments**.** Scale bar: 1000µm. Quantification of transwell migration of control or *CEBPA* knock-down MCF10A cells is shown. The number of cells present in the bottom of the transwell was quantified after 24 hours incubation. Data was quantified relative to scrambled control cells and is represented as mean ± SD of n=24 independent experiments. p-values were calculated using unpaired two-tailed Student's t-test. *$p < 0.05$.

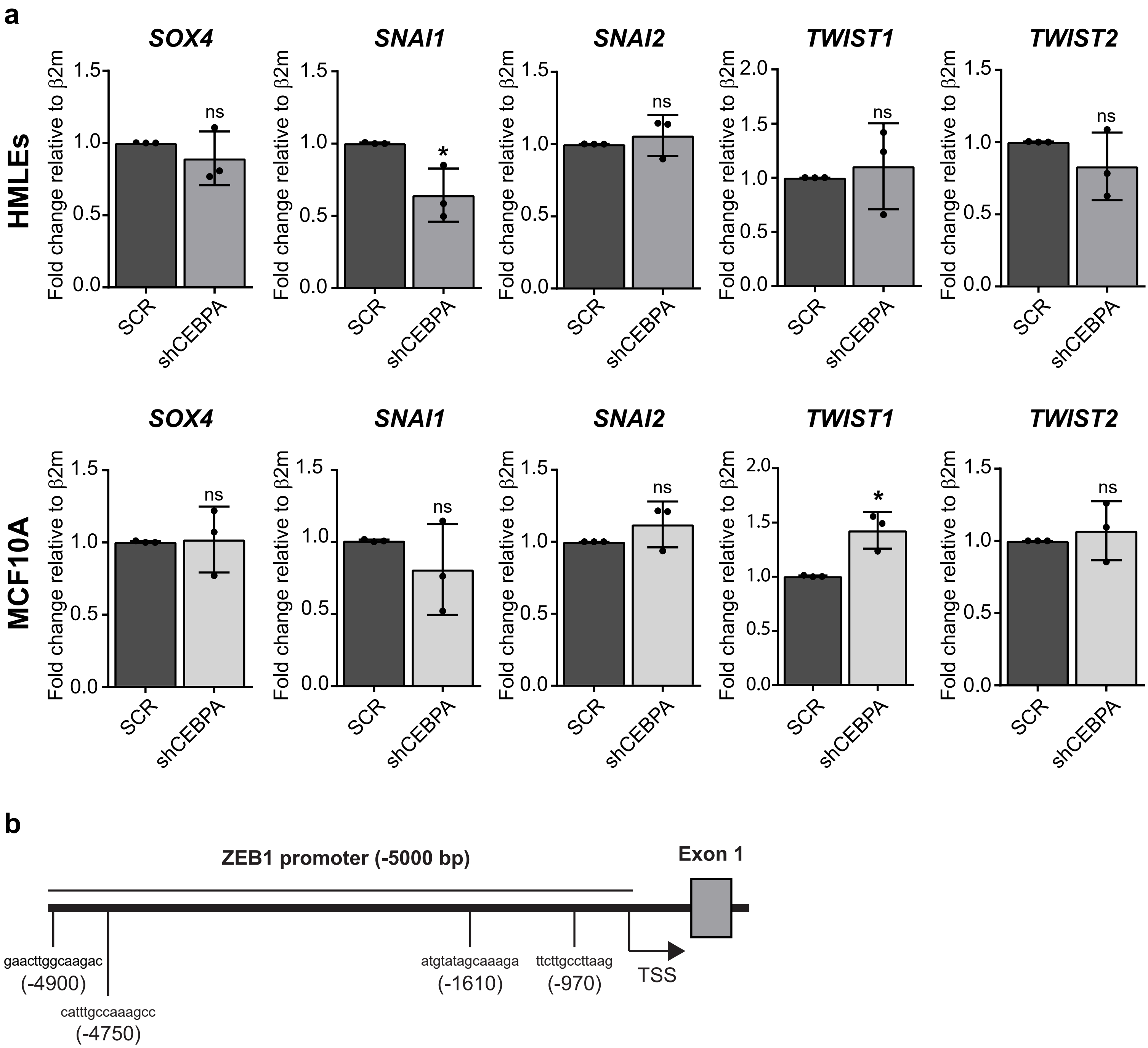

Supplementary Figure 5. The effect of CEBPA knock-down on EMT-effectors. (a) qRT-PCR results displaying the effect of CEBPA knock-down on the expression of known EMT-TFs both in HMLE and MCF10A cells. Data represented as mean ± SD of three independent experiments. p-values were calculated using unpaired two-tailed Student's t-test. *p < 0.05. ns: not significant. **(b)** Schematic representation of the *ZEB1* promoter region and predicted C/EBPα binding sites using Contra V2 software and high stringent parameters (core 0.95; similarity matrix 0.85).

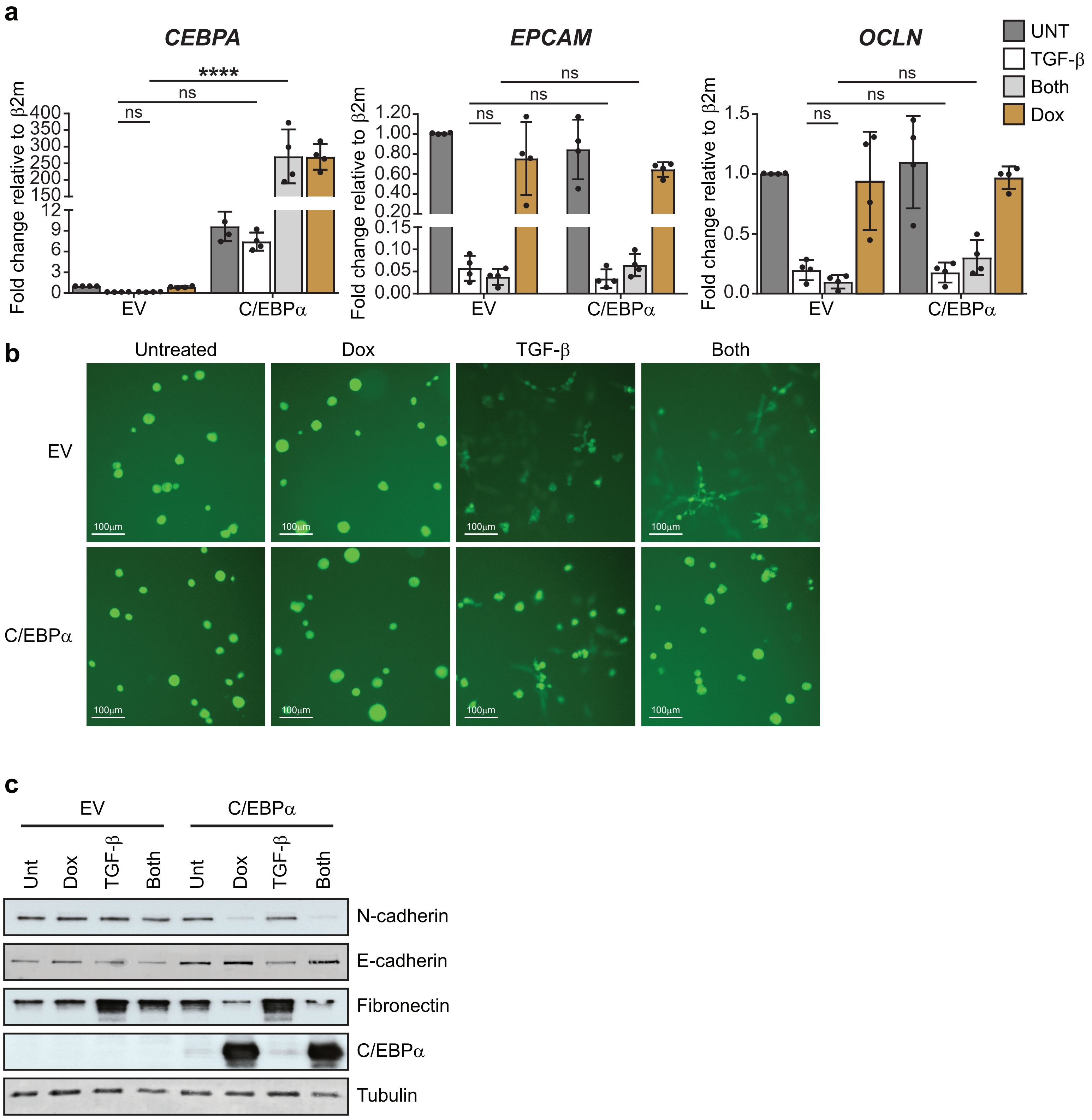

Supplementary Figure 6. Impact of doxycycline-inducible CEBPA on MCF10A-derived epithelial-spheroids treated with TGF-β. (a) Results from qRT-PCR showing the expression levels of *CEBPA*, *EPCAM* and *OCLN* in EV or CEBPA-inducible HMLE cells treated with TGF-β, doxycycline or both. Data represented as mean ± SD of four independent experiments. p-values were calculated using two-way ANOVA with Tukey's multiple comparisons test. ****$p<0.0001$. ns: not significant. **(b)** Fluorescence microscopy visualization of the epithelial-spheroids formed in MCF10A cells expressing empty vector or doxycycline-inducible CEBPA upon TGF-β, doxycycline or both. Scale bar: 100µm. Data is representative of three independent experiments. **(c)** Immunoblotting analysis of EV or CEBPA-inducible MCF10A cells treated with TGF-β, doxycycline or both. Data is representative of three independent experiments.

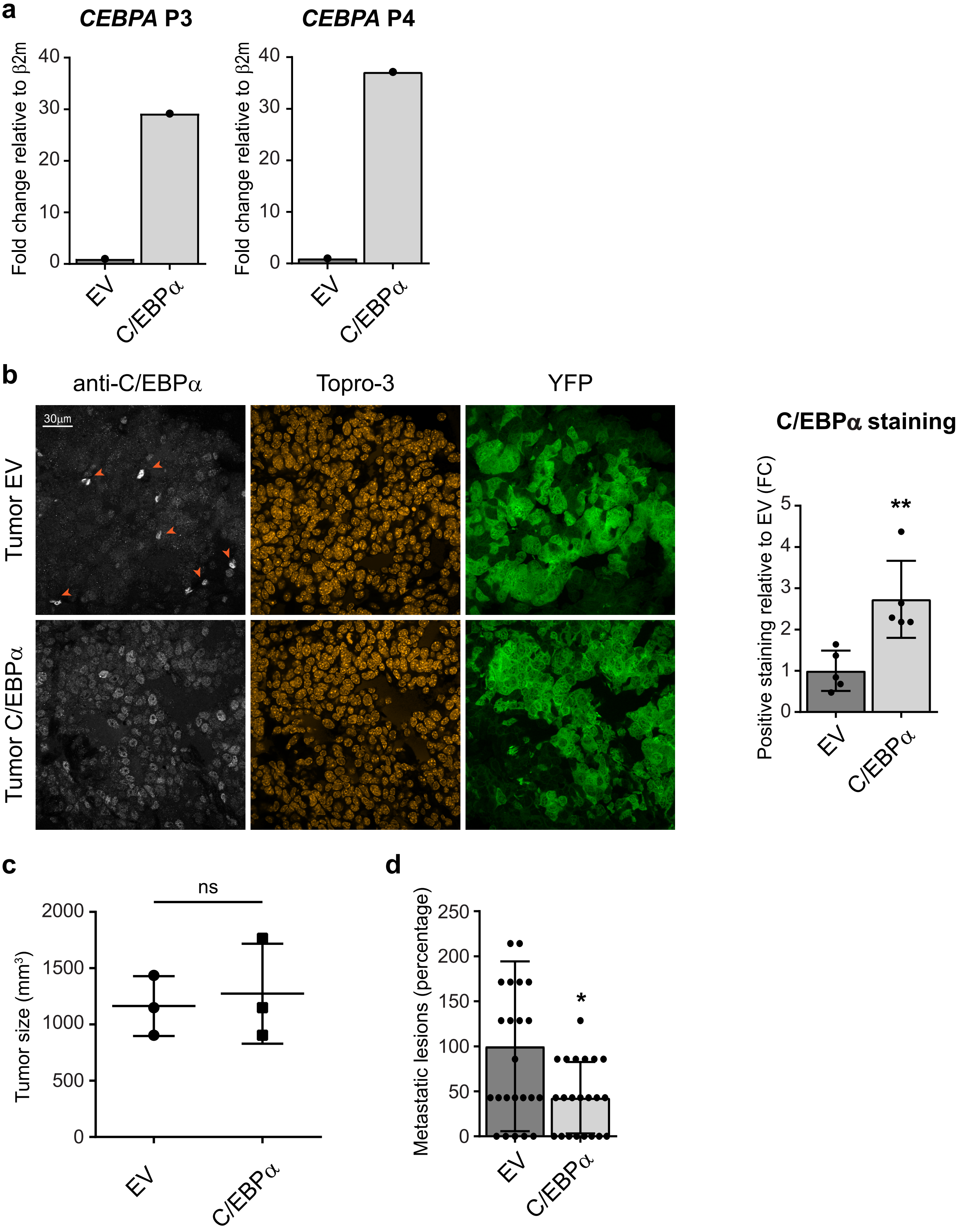

Supplementary Figure 7. C/EBPα expression levels in the tumor organoids and primary tumors. **(a)** qRT-PCR analysis of *CEBPA* expression in control or C/EBPα-overexpressing tumor organoids used for the injection into NGS mice. Two independent set of primers were used (P3 and P4). **(b)** Immunofluorescence images and quantification of primary mammary tumors of control or C/EBPα-mice stained for C/EBPα. Arrowheads indicate cells outside the primary tumor that endogenously express C/EBPα. Data is representative of five mice per group. p-values were calculated using unpaired two-tailed Student's t-test. $^{**}p < 0.01$. Scale bar: 30µm. **(c)** Tumor volume of primary mammary tumors at mastectomy day. n =3 per experimental group. p-values were calculated using paired two-tailed Student's t-test. ns: not significant. **(d)** Quantification of the number of lung metastasis nodules from mice injected with control or C/EBPα-overexpressing tumor organoids. n=3 per experimental group with eight different sections per lung. p-values were calculated using unpaired two-tailed Student's t-test. $^{*}p < 0.05$.

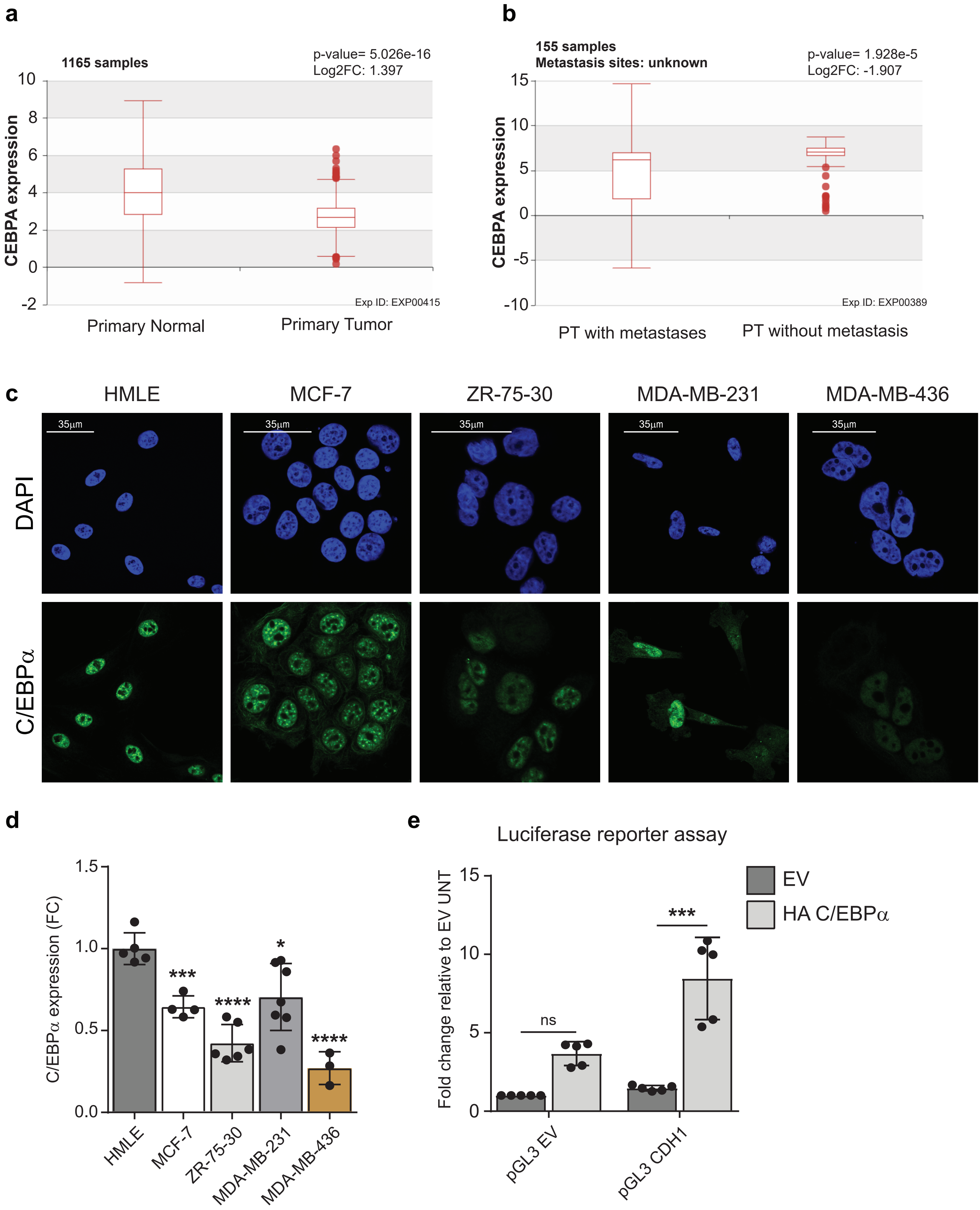
a
1165 samples
p-value= 5.026e-16
Log2FC: 1.397
CEBPA expression
Exp ID: EXP00415
Primary Normal
Primary Tumor
b
155 samples
Metastasis sites: unknown
p-value= 1.928e-5
Log2FC: -1.907
CEBPA expression
Exp ID: EXP00389
PT with metastases
PT without metastasis
c
HMLE
MCF-7
ZR-75-30
MDA-MB-231
MDA-MB-436
DAPI
C/EBPα
35μm
d
C/EBPα expression (FC)
***
****
*
****
HMLE
MCF-7
ZR-75-30
MDA-MB-231
MDA-MB-436
e
Luciferase reporter assay
Fold change relative to EV UNT
EV
HA C/EBPα
ns
***
pGL3 EV
pGL3 CDH1

Supplementary Figure 8. Clinical relevance of C/EBPα expression in breast cancer. (a) *CEBPA* expression in normal breast tissue compared to primary tumor tissue and **(b)** *CEBPA* expression in primary tumor with metastasis compared to primary tumor without metastasis of breast cancer patients obtained by analyzing the public available database HCMDB (Human Cancer Metastasis Database). Exp ID: EXP00415, Primary Normal: upper 5.268, lower 0, median 2.832, Q3 4.003 and Q1 -0.822; Primary Tumor: upper 3.164, lower 1, median 2.133, Q3 2.676 and Q1 0.586, Exp ID: EXP00389, PT with metastases: upper 6.967, lower 0, median 1.838, Q3 6.190 and Q1 -5.856; PT without metastases: upper 7.486, lower 1, median 6.660, Q3 7.038 and Q1 5.422. Statistical significance was calculated using differential expression analysis. **(c)** Immunofluorescence staining and **(d)** quantification of C/EBPα expression levels in several human breast cancer cell lines compared to untransformed HMLE cells. Data is representative of n=3-7 independent experiments per group. p-values were calculated using unpaired two-tailed Student's t-test. *p < 0.05, ***p < 0.001 and ****p<0.0001. Scale bar: 35µm. (e) Luciferase assay in HEK293T cells transiently transfected with pGL3 empty vector or pGL3 containing *CDH1* promoter and co-transfected with pcDNA3 empty vector or pcDNA3 HA C/EBPα. Results obtained from five independent biological replicates wherein three independent technical replicates were used per condition. Results are normalized to renilla values. p-values were calculated using two-way ANOVA with Tukey's multiple comparisons test. ***p<0.001. ns: not significant.

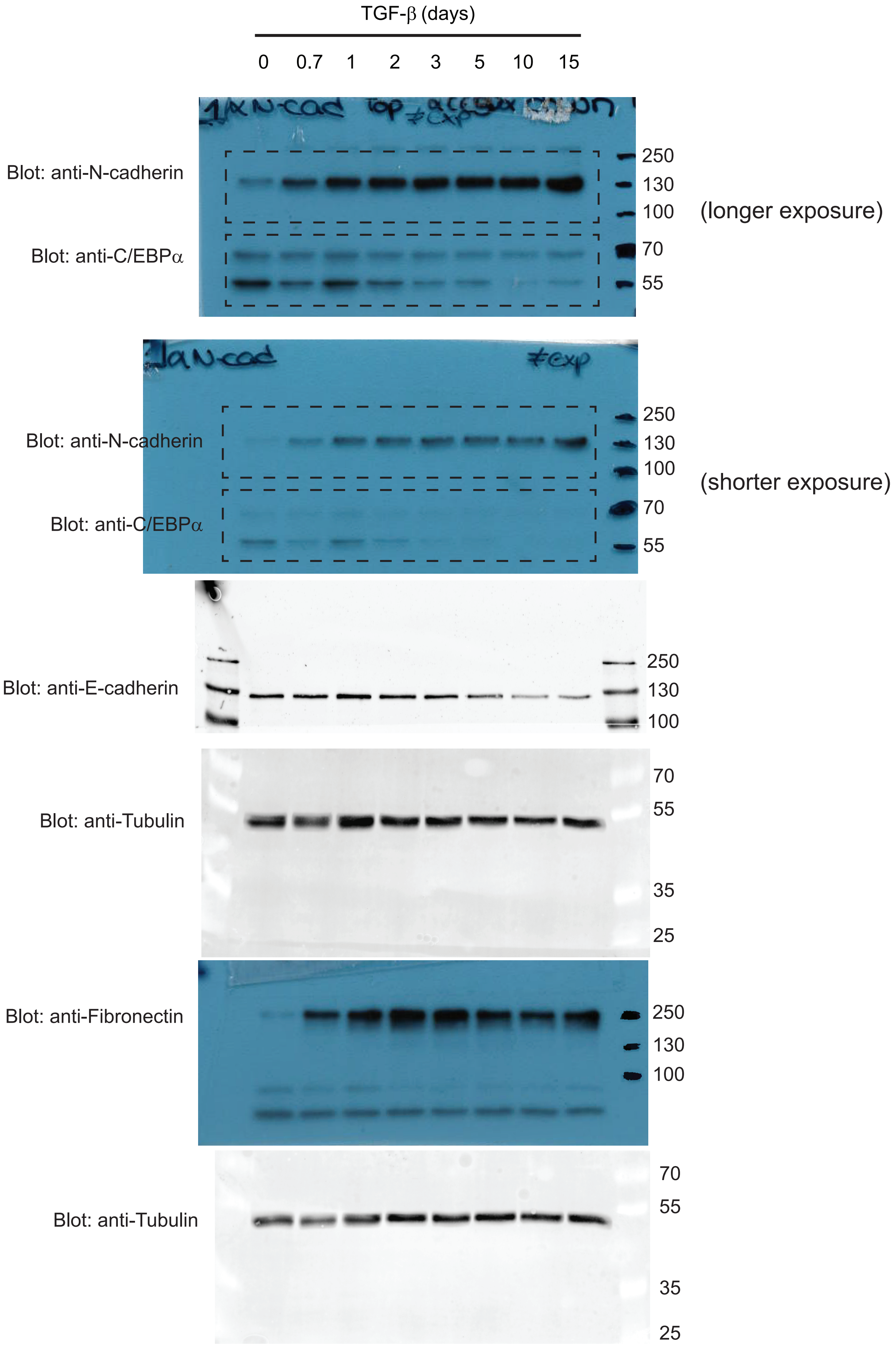

Supplementary Figure 9. Western blot results of the effect of TGF-β treatment on HMLE cells (Figure 1d).

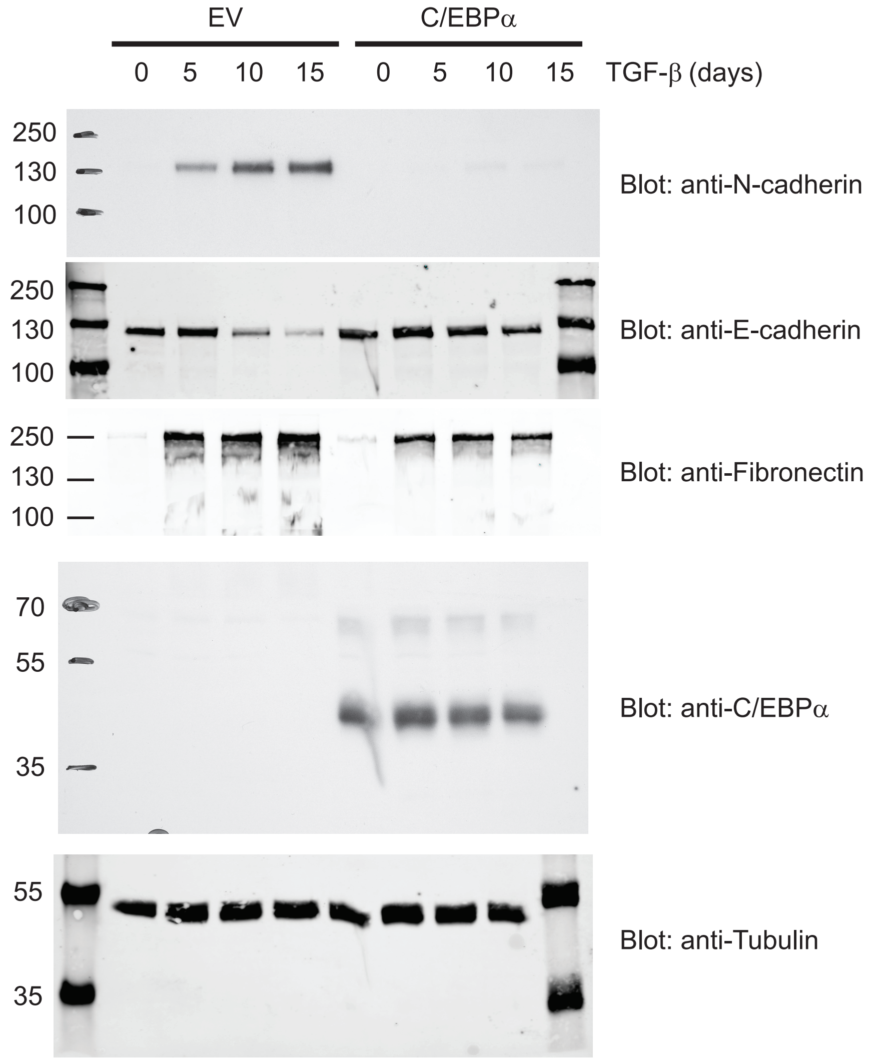

Supplementary Figure 10. Western blot results of the effect of C/EBPα overexpression on TGF-b-mediated EMT (Figure 4c).

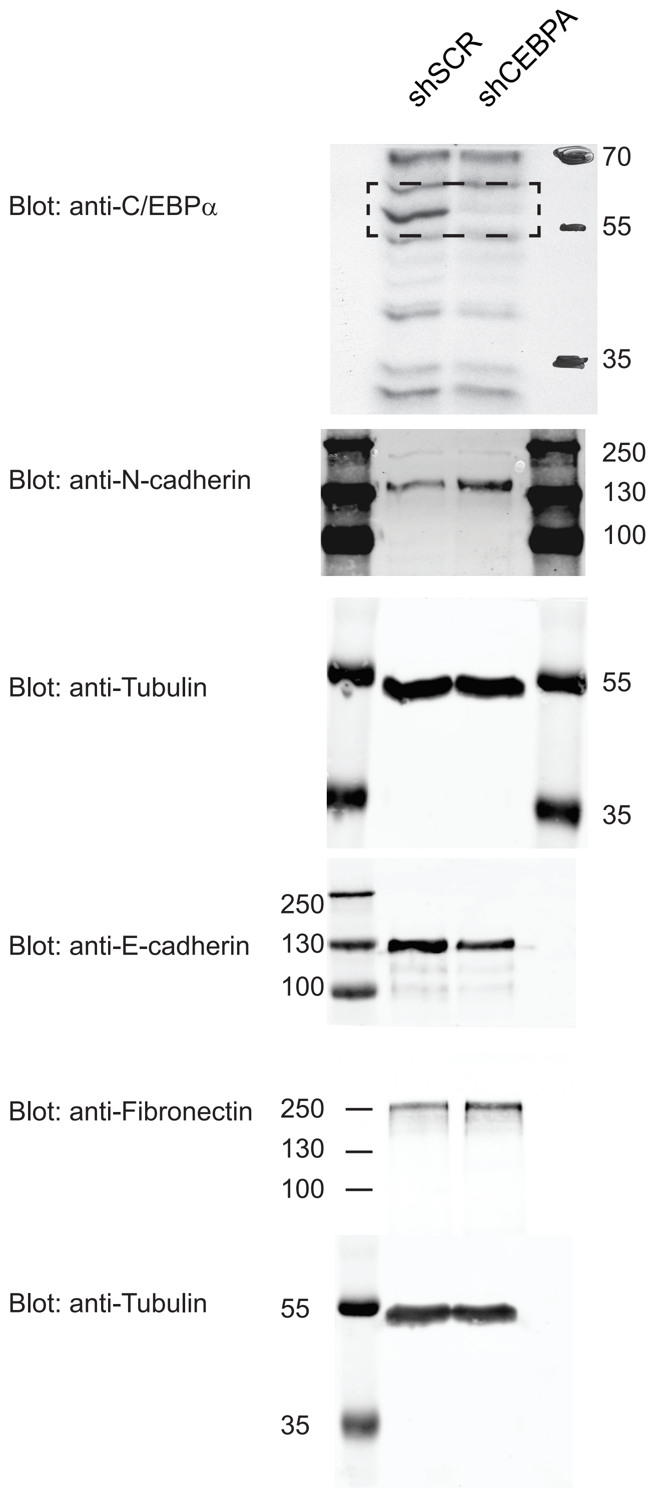

Supplementary Figure 11. Western blot results of the effect of C/EBPα knock-down on EMT markers in HMLE cells (Figure 5d).

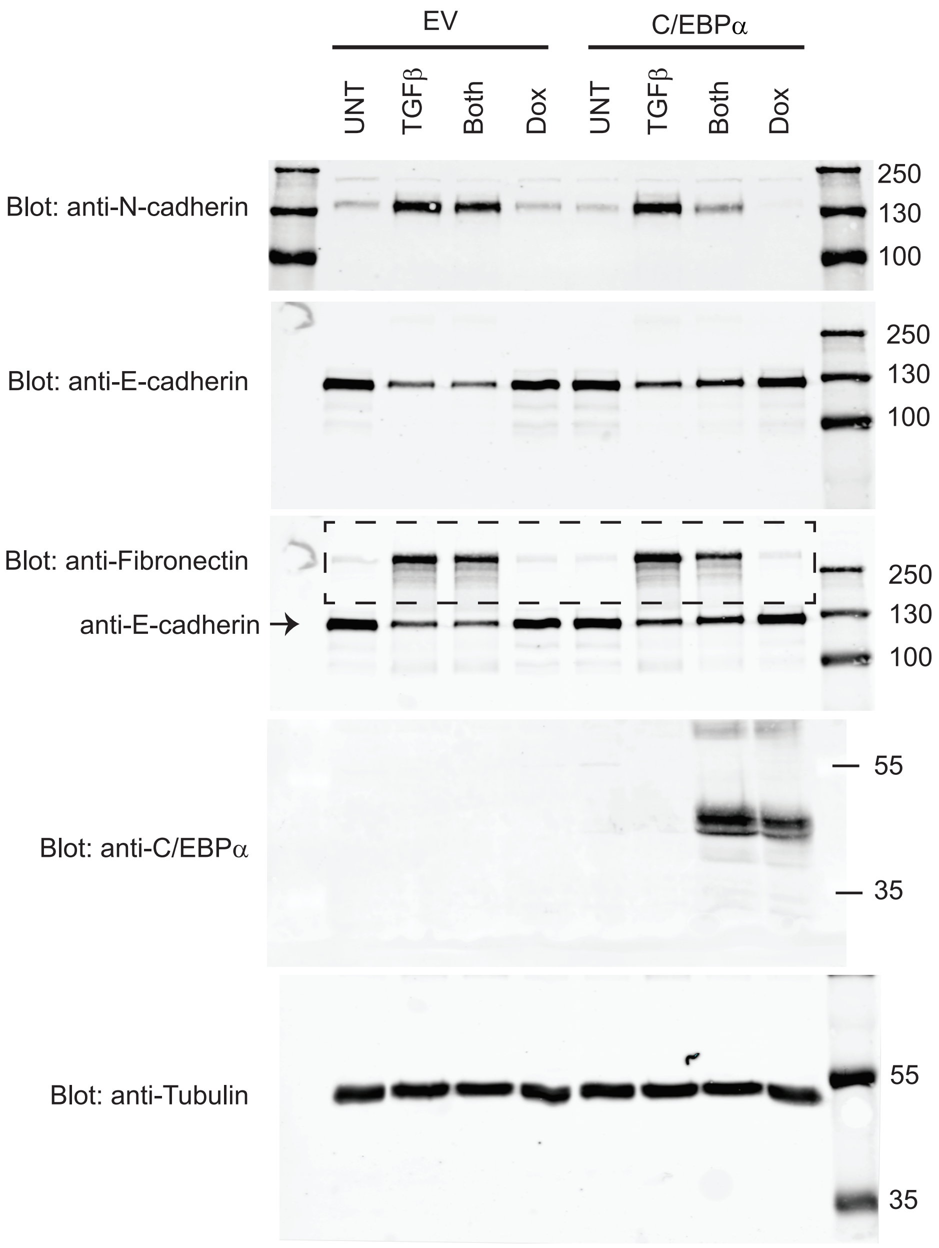

Supplementary Figure 12. Western blot results of the effect of C/EBPα conditional activation on EMT (Figure 6c).

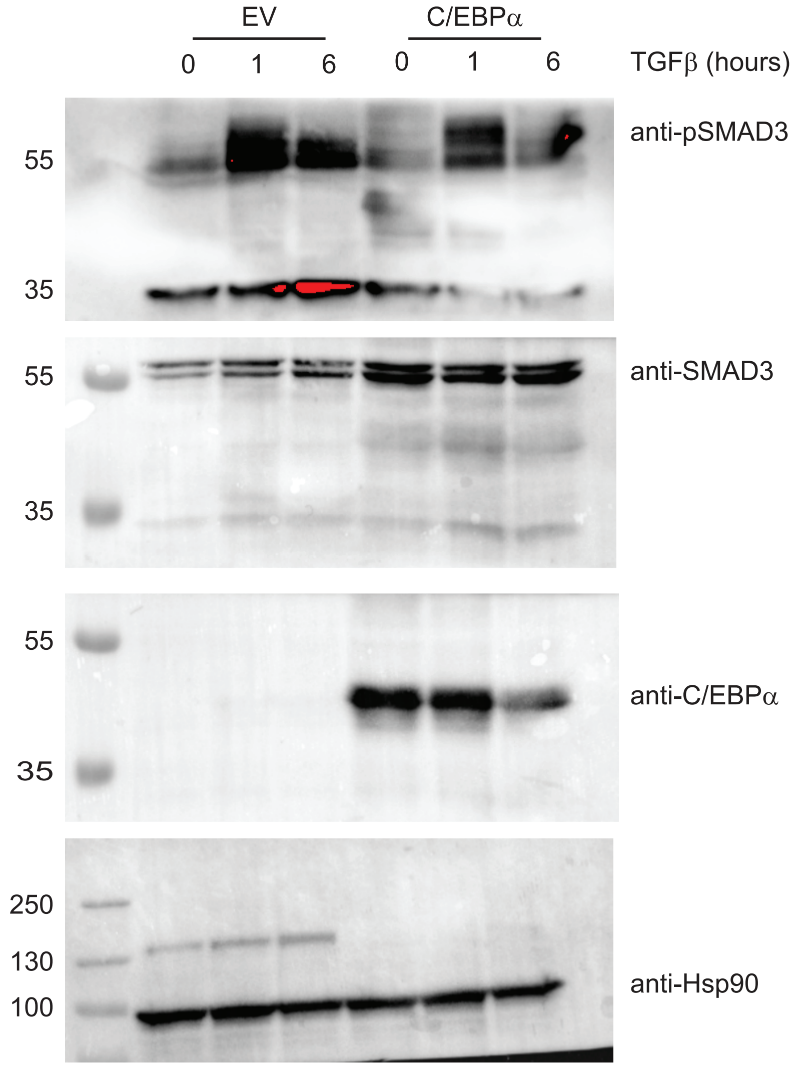

Supplementary Figure 13. Western blot results of the effect of C/EBPα overexpression on Smad3 activity during TGF-b treatment (Supplementary Figure 3c).

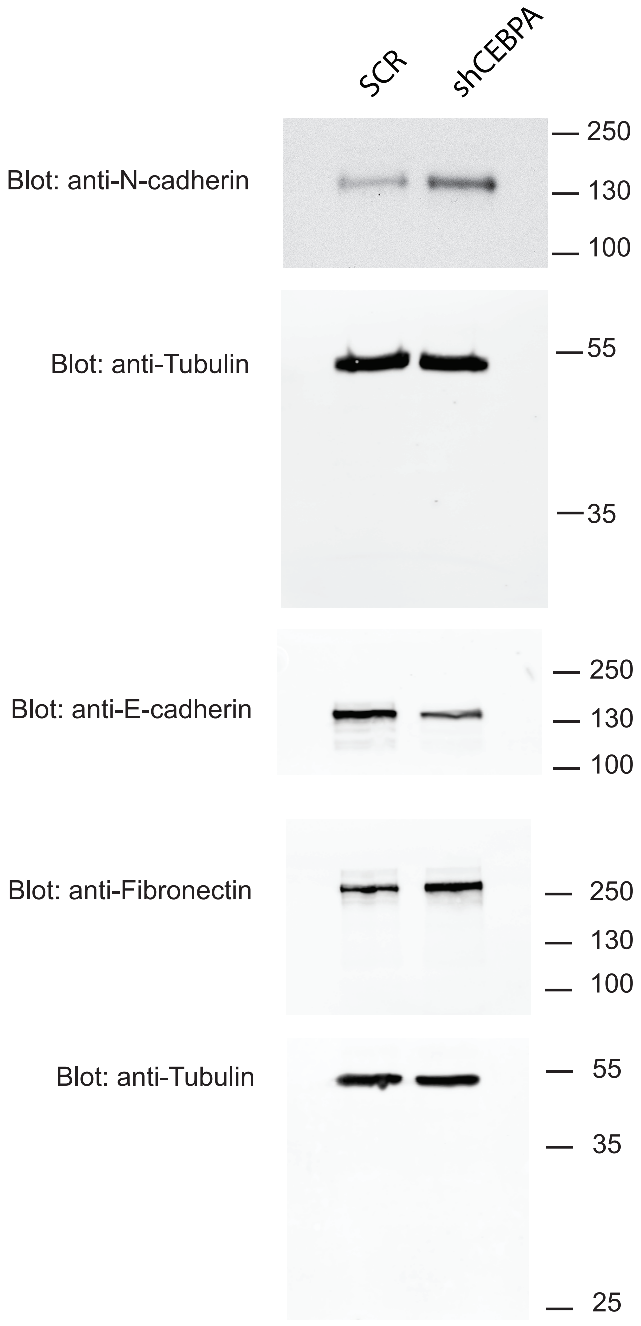

Supplementary Figure 14. Western blot results of the effect of C/EBPα knock-down on EMT markers in MCF10A cells (Supplementary Figure 4e).

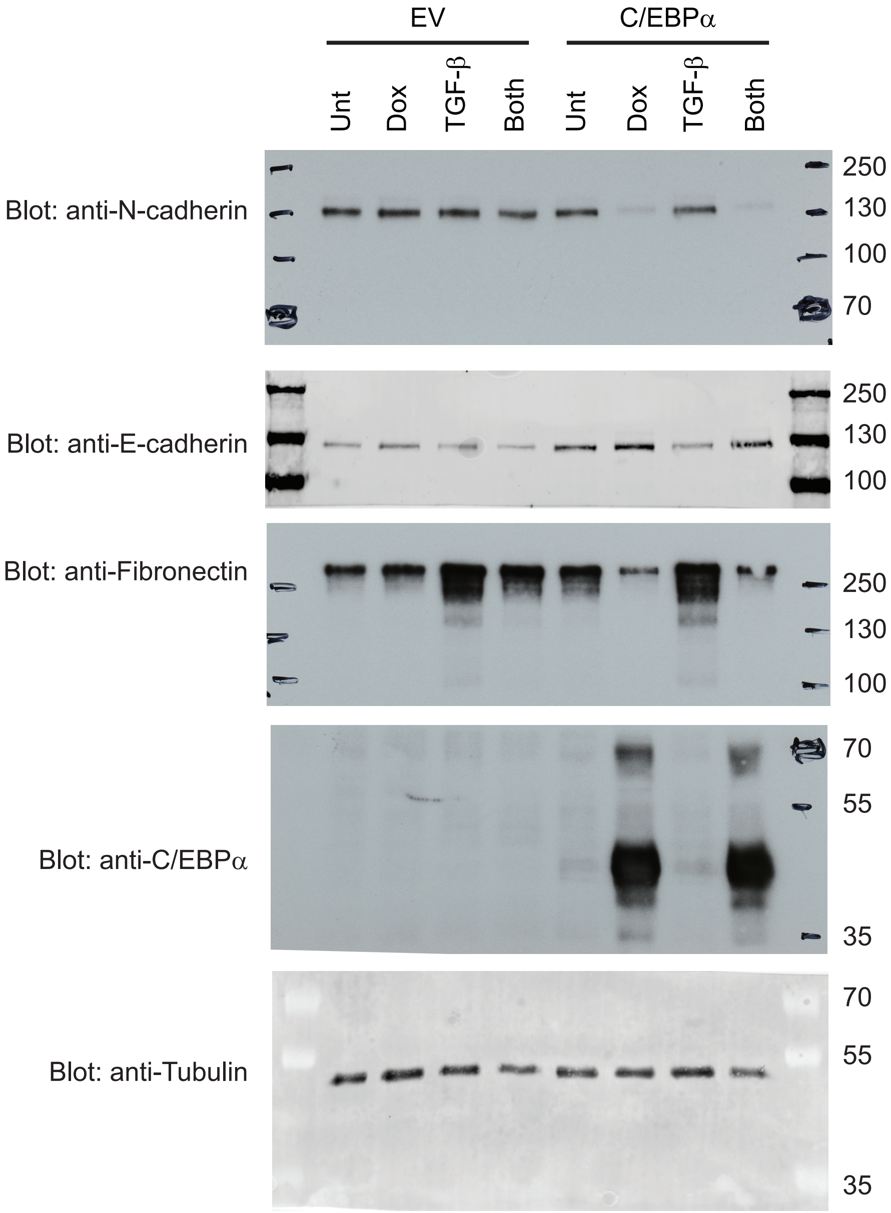

Supplementary Figure 15. Western blot results of the effect of C/EBPα conditional activation on EMT in MCF10A cells (Supplementary Figure 6c).
