# Supplementary material for: C/EBPɑ is crucial determinant of epithelial maintenance by preventing epithelial-to-mesenchymal transition
Source: Nat Commun. 2020 Feb 7;11:785. doi: 10.1038/s41467-020-14556-x (PMC7005738; doi:10.1038/s41467-020-14556-x)
Supplement: Supplementary file 3 — Reporting Summary [file 41467_2020_14556_MOESM3_ESM.pdf]

nature research

Corresponding author(s): Paul J. Coffer

Last updated by author(s): Jan 14, 2020

Reporting Summary

Nature Research wishes to improve the reproducibility of the work that we publish. This form provides structure for consistency and transparency in reporting. For further information on Nature Research policies, see Authors & Referees and the Editorial Policy Checklist.

Statistics

For all statistical analyses, confirm that the following items are present in the figure legend, table legend, main text, or Methods section.

n/a	Confirmed	
☐	☒	The exact sample size (n) for each experimental group/condition, given as a discrete number and unit of measurement
☐	☒	A statement on whether measurements were taken from distinct samples or whether the same sample was measured repeatedly
☐	☒	The statistical test(s) used AND whether they are one- or two-sided<br>*Only common tests should be described solely by name; describe more complex techniques in the Methods section.*
☐	☒	A description of all covariates tested
☐	☒	A description of any assumptions or corrections, such as tests of normality and adjustment for multiple comparisons
☐	☒	A full description of the statistical parameters including central tendency (e.g. means) or other basic estimates (e.g. regression coefficient) AND variation (e.g. standard deviation) or associated estimates of uncertainty (e.g. confidence intervals)
☐	☒	For null hypothesis testing, the test statistic (e.g. F, t, r) with confidence intervals, effect sizes, degrees of freedom and P value noted<br>*Give P values as exact values whenever suitable.*
☒	☐	For Bayesian analysis, information on the choice of priors and Markov chain Monte Carlo settings
☒	☐	For hierarchical and complex designs, identification of the appropriate level for tests and full reporting of outcomes
☒	☐	Estimates of effect sizes (e.g. Cohen's d, Pearson's r), indicating how they were calculated

Our web collection on statistics for biologists contains articles on many of the points above.

Software and code

Policy information about availability of computer code

Data collection	N/A
Data analysis	N/A

For manuscripts utilizing custom algorithms or software that are central to the research but not yet described in published literature, software must be made available to editors/reviewers. We strongly encourage code deposition in a community repository (e.g. GitHub). See the Nature Research guidelines for submitting code & software for further information.

Data

Policy information about availability of data

All manuscripts must include a data availability statement. This statement should provide the following information, where applicable:

- Accession codes, unique identifiers, or web links for publicly available datasets
- A list of figures that have associated raw data
- A description of any restrictions on data availability

Data presented on figures 2a and S1c were generated by analyzing the data available under the accession numbers GSE104761 and GSE24202, respectively. The datasets generated during the current study (RNA-seq) were deposited under the accession number GSE143612. Uncropped western blot Images can be found in the supplementary information section (Supplementary Figures 9-15).

Field-specific reporting

Please select the one below that is the best fit for your research. If you are not sure, read the appropriate sections before making your selection.

☒ Life sciences ☐ Behavioural & social sciences ☐ Ecological, evolutionary & environmental sciences

For a reference copy of the document with all sections, see nature.com/documents/nr-reporting-summary-flat.pdf

Life sciences study design

All studies must disclose on these points even when the disclosure is negative.

Sample size	For in vitro and in vivo studies, at least three biological replicates comprised our analysis. Except for western blot analysis, at least two technical replicates per condition were performed. For in vivo studies, 3-5 animals per group as the minimal number of animals used in order to detect a difference of statistical and biological significance.
Data exclusions	No data were excluded from the analyses.
Replication	All attempts at replication were successful.
Randomization	Confocal images were acquired from random and unbiased areas of the cover slips. Cell lines and animals used were equally healthy and exposed to same conditions before the experiment.
Blinding	Quantification of micro and macrometastases of the lungs of the animals were performed by two independent scientists and blinded.

Reporting for specific materials, systems and methods

We require information from authors about some types of materials, experimental systems and methods used in many studies. Here, indicate whether each material, system or method listed is relevant to your study. If you are not sure if a list item applies to your research, read the appropriate section before selecting a response.

Materials & experimental systems

n/a	Involved in the study	
☐	☒	Antibodies
☐	☒	Eukaryotic cell lines
☒	☐	Palaeontology
☐	☒	Animals and other organisms
☒	☐	Human research participants
☒	☐	Clinical data

Methods

n/a	Involved in the study	
☒	☐	ChIP-seq
☒	☐	Flow cytometry
☒	☐	MRI-based neuroimaging

Antibodies

Antibodies used	The following primary antibodies were used for WB: anti-C/EBPα (Santa Cruz Biotechnology, sc-61), anti-N-cadherin (BD Biosciences, 610921), anti-E-cadherin (BD Biosciences, 610182), anti-Fibronectin (BD Biosciences, 610077), anti-α-tubulin (Sigma, T90026). For immunofluorescence analysis we used anti-C/EBPα (abcam, ab128482), anti-E-cadherin (abcam, ab1416) and anti-Fibronectin (BD Biosciences, 610077). For immunofluorescence labeling of frozen sections, anti-C/EBPα (Cell Signaling Technology, 2295) was used.
Validation	Most of the antibodies used in this study were validated previously in our laboratory for previous and published studies. C/EBPα antibody (sc-61) was validated firstly by performing WB analysis using myeloid cells in different differentiated states to confirm the molecular weight of endogenous C/EBPα. After and using C/EBPα knockdown cells, we confirmed that band with the expected pattern disappeared. For confocal imaging, IgG control was used to validate both C/EBPα antibodies.

Eukaryotic cell lines

Policy information about cell lines

Cell line source(s)	MCF10A cells were kindly provided by Dr. Patrick Derksen and HMLE cells were kindly provided by Prof. Dr. Robert Weinberg. HEK293 cells for virus production and breast cancer cell lines were acquired from ATCC.
Authentication	The phenotype and molecular markers of all cell lines were carefully checked before assays and analyses.
Mycoplasma contamination	All cell lines tested negative for mycoplasma contamination.

Commonly misidentified lines (See ICLAC register)	No misidentified cells lines were used in this study.

Animals and other organisms

Policy information about studies involving animals; ARRIVE guidelines recommended for reporting animal research

Laboratory animals	Non-obese diabetic SCID IL-2 receptor gamma chain knockout (NSG) mice were used in this study. All female and around 8 weeks old.
Wild animals	This study did not involve wild animals.
Field-collected samples	This study did not involve samples collected from the field
Ethics oversight	All experiments were carried out in accordance with the guidelines of the Animal Welfare Committee of the Royal Netherlands Academy of Arts and Sciences, The Netherlands.

Note that full information on the approval of the study protocol must also be provided in the manuscript.
